# Advancing the Utility of Low-Vision Aids in the Rehabilitation of Vision in Unclassified Macular Dystrophy: A Report of a Rare Disease

**DOI:** 10.7759/cureus.109228

**Published:** 2026-05-19

**Authors:** Selva Seelan Samuel, Samuel Livingstone Kumaran, Somreeta Bhattacharya, Peachiyammal Veerapandian, Radha Annamalai

**Affiliations:** 1 Department of Optometry, Faculty of Allied Health Sciences, Sri Ramachandra Institute of Higher Education and Research, Chennai, IND; 2 Department of Ophthalmology, Sri Ramachandra Institute of Higher Education and Research, Chennai, IND

**Keywords:** autofluorescence, directory reader, low-vision aids, macular dystrophy, perimetry

## Abstract

Macular dystrophies are a group of inherited retinal disorders that primarily affect the macula, leading to progressive loss of central vision. This case report describes a 14-year-old girl who has had difficulty with reading, writing, and face recognition in both eyes for four years. The past medical history includes treatment for a small apical muscular ventricular septal defect in 2015. There was no history of ocular surgery or trauma. Her initial unaided visual acuity was 1.0 logMAR in both eyes for distance and N24 at 15-20 cm in both eyes for near vision, assessed using a vocational near-vision chart. Fundus examination revealed retinal pigment epithelium (RPE) alterations, scattered flecks, and vascular anomalies with atypical atrophy in the macula in both eyes, which did not conform to the established macular dystrophies that are described in routine practice. A diagnosis of unclassified macular dystrophy was made. Low-vision aids, including a directory reader, high-add lenses, and a monocular telescope, along with eccentric viewing training, were prescribed. This resulted in an improvement in her functional vision, thereby providing opportunities to enhance her quality of life. The objective of this case report was to highlight expanding presentations of both typical and atypical macular dystrophy and the importance of low-vision aids in the rehabilitation of these patients.

## Introduction

Macular dystrophies are a group of inherited retinal disorders characterized by the progressive degeneration of the macula, leading to loss of central vision [1]. It predominantly affects central vision, which is responsible for high-resolution visual tasks such as reading, writing, and facial recognition [2,3]. Pediatric macular dystrophy comprises an established group that includes Stargardt disease, Best’s vitelliform retinal dystrophy, and juvenile retinoschisis [4]. Other dystrophies with unrecognized features that do not fit into conventional categories are increasingly being identified in clinical practice. The most common presentations of macular dystrophy include decreased visual acuity and color vision, photophobia, central vision loss or scotomas in the visual field, amblyopia, nystagmus, and strabismus. Pediatric macular disorders present with central vision loss and difficulty with fine visual tasks, such as reading and recognizing faces, underscoring their impact on quality of life and functional outcomes in children. Early onset of the disease can significantly impair academic performance and quality of life, necessitating a tailored and comprehensive approach to management [5,6].

Macular dystrophies can affect a child's visual development and educational outcomes, primarily due to the loss of central vision, which is often associated with the loss of binocular single vision (BSV). This can affect fine motor skills, reading abilities, and visual attention, potentially delaying learning and cognitive development [7]. Additionally, varying degrees of visual impairment can have profound psychological and social effects, increasing the likelihood of social isolation, anxiety, and lower self-esteem compared to peers. Children with visual impairments frequently face academic challenges, highlighting the need for personalized educational support and interventions [8].

Low-vision devices play a crucial role in managing conditions such as macular dystrophy by enhancing residual vision, improving functional independence, and supporting educational and daily activities. Early use of low-vision aids, such as magnifiers, high-add lenses, and telescopes, can help maximize remaining vision and enhance functional capabilities [9]. Adaptive strategies, such as training in eccentric viewing to utilize peripheral vision, can further improve visual performance. Hence, the primary purpose of this case report was to emphasize the possibility of atypical macular dystrophy and to reiterate the importance of low-vision rehabilitation in optimizing residual vision, fostering independence in daily activities, and promoting overall well-being.

## Case presentation

A 14-year-old girl presented with complaints of difficulty in reading, writing, and recognizing faces in both eyes for the past four years. She had a history of apical muscular ventricular septal defect diagnosed in 2015, for which she received appropriate treatment. The ocular diagnosis was established one year before, and low-vision rehabilitation was initiated within one month of diagnosis.

She reported significant difficulty with near vision, particularly affecting reading and her school activities. On examination, unaided distance visual acuity was 1.0 logMAR in both eyes. Unaided near visual acuity was N24 at 15-20 cm in both eyes, assessed using a continuous text (vocational near vision) chart. Anterior segment evaluation was unremarkable, with brisk pupillary reactions, and intraocular pressure was 13 mmHg in both eyes using a non-contact tonometer.

**Figure 1:**
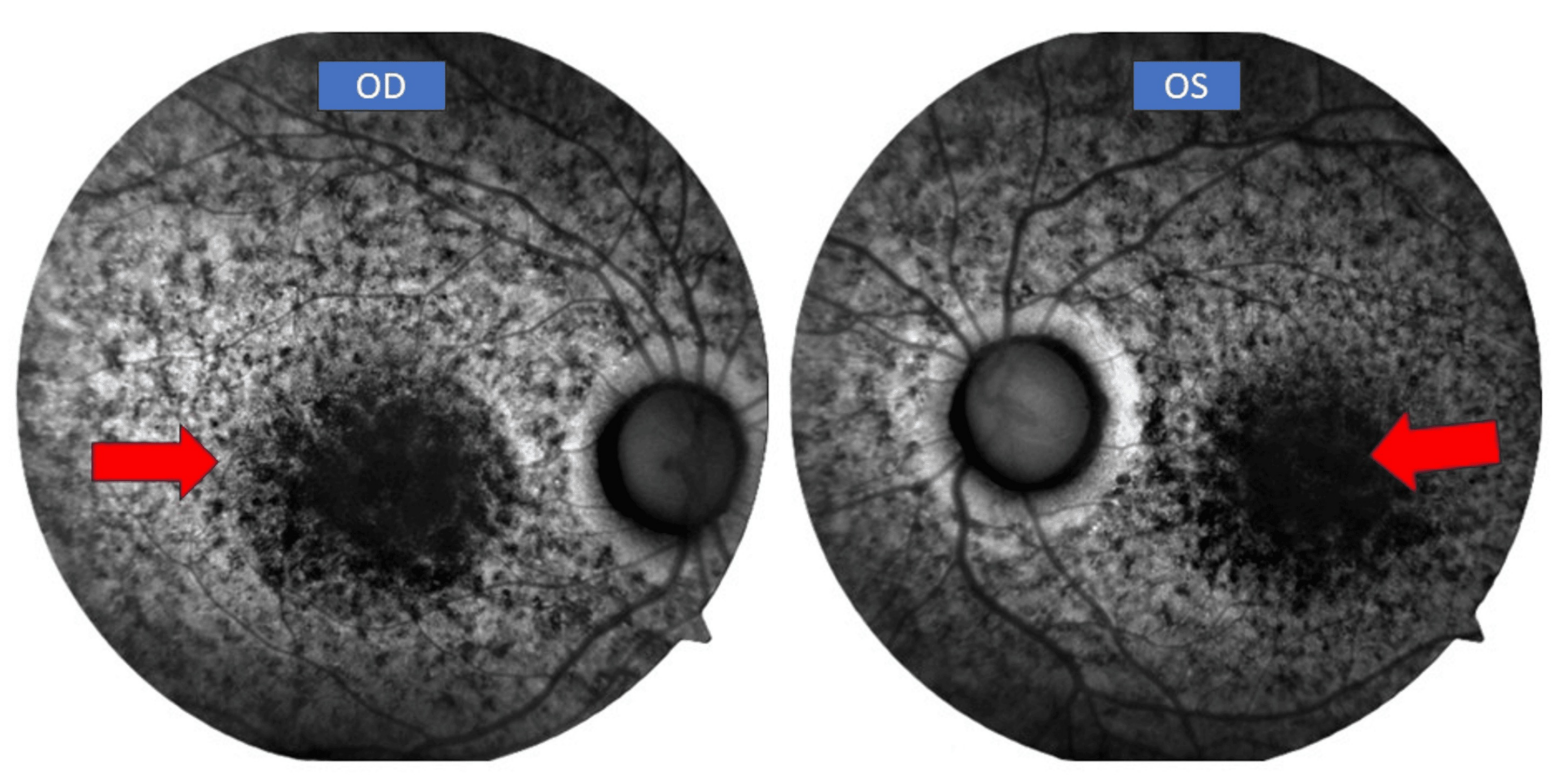
Fundus autofluorescence shows hyperautofluorescence at macula.

**Figure 2:**
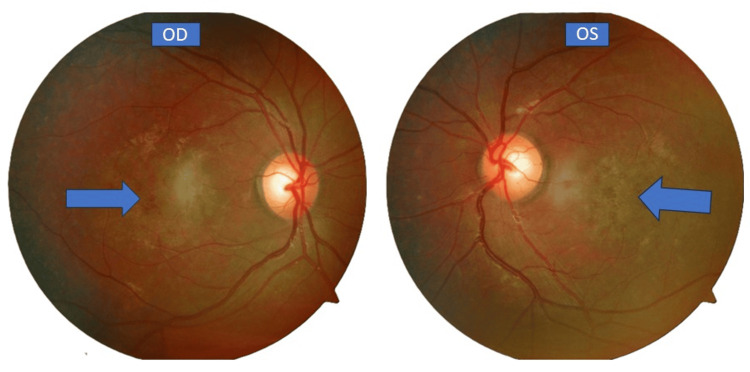
Color fundus photo shows bilateral symmetric atrophic areas and scattered flecks in the macula.

**Figure 3:**
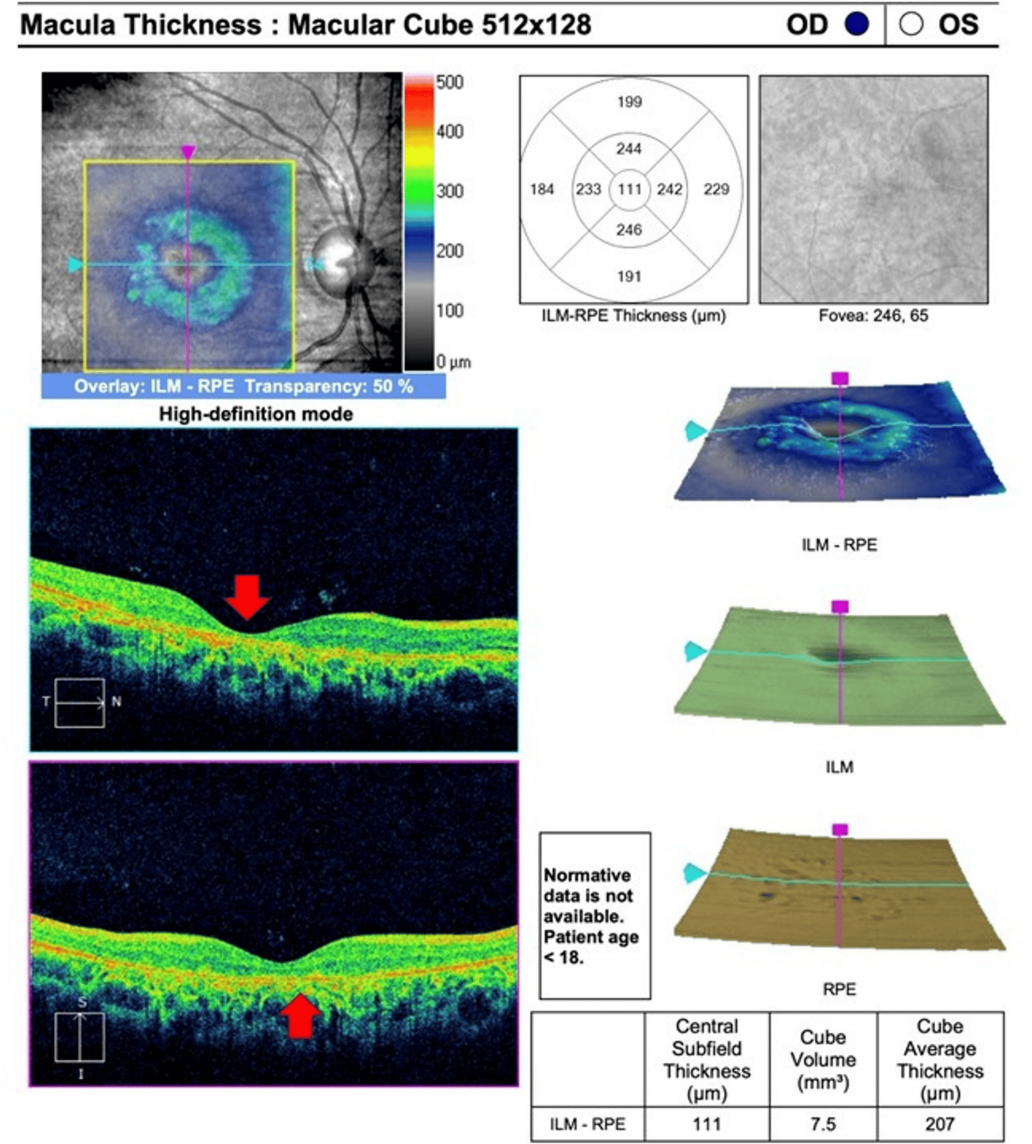
OCT revealed thinning of the fovea with disorganization of the retinal pigment epithelium in right eye. ILM: inner limiting membrane; RPE: retinal pigment epithelium; OCT: optical coherence tomography

**Figure 4:**
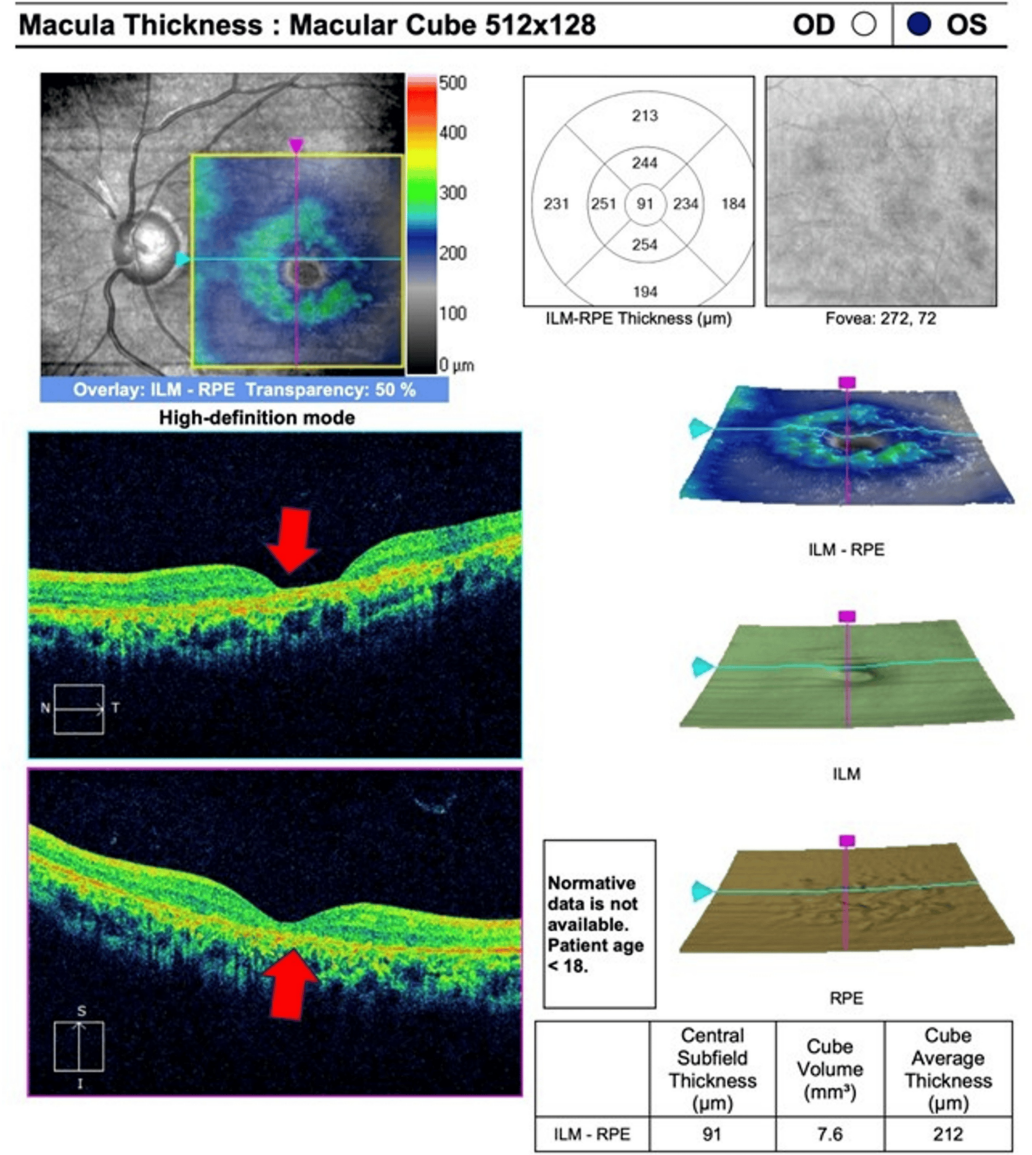
OCT revealed thinning of the fovea with disorganization of the retinal pigment epithelium in left eye. ILM: inner limiting membrane; RPE: retinal pigment epithelium; OCT: optical coherence tomography

**Figure 5:**
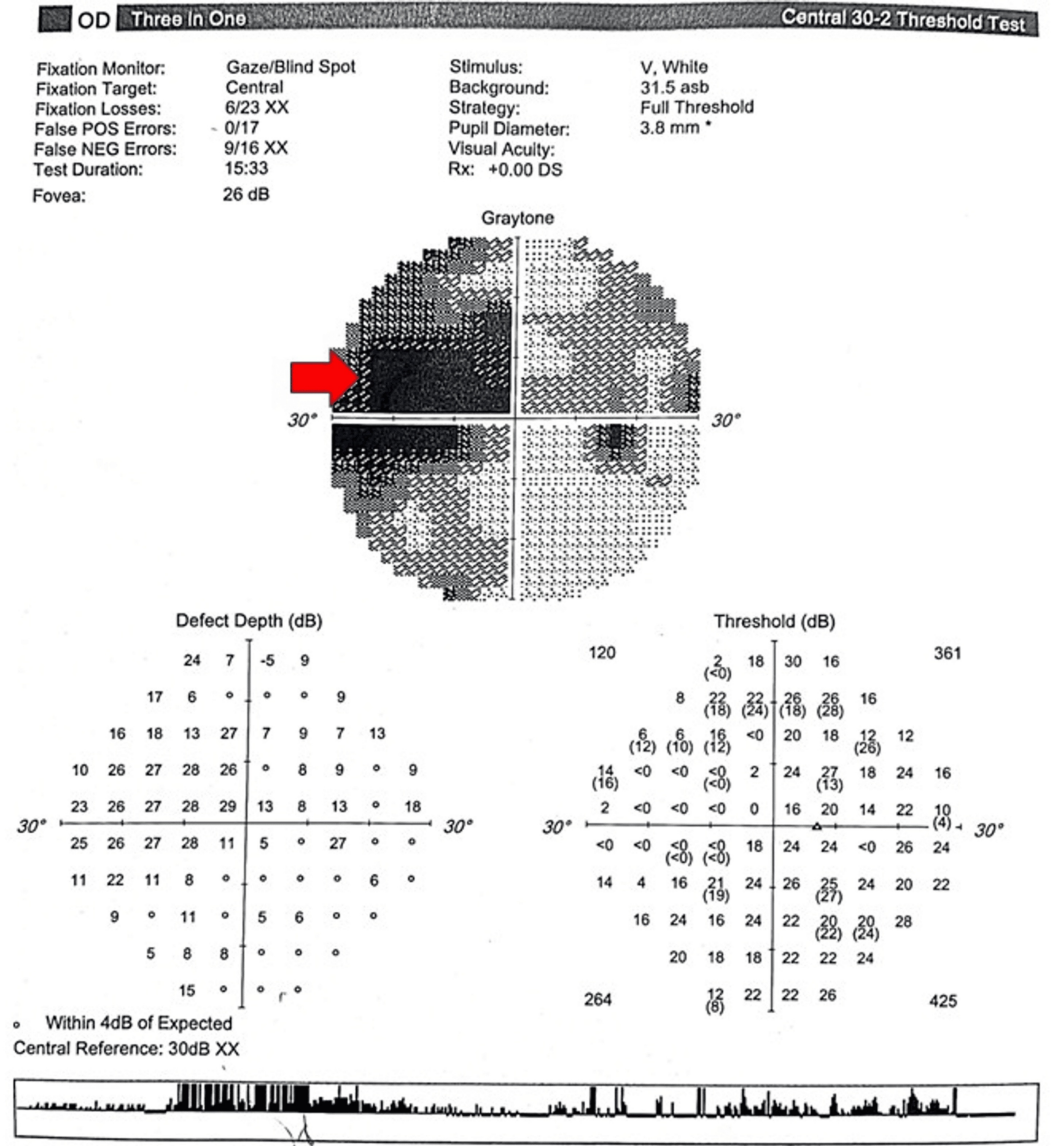
Humphrey Visual Field test showed depression in sensitivity in right eye. POS: false positive error; NEG: false negative error

**Figure 6:**
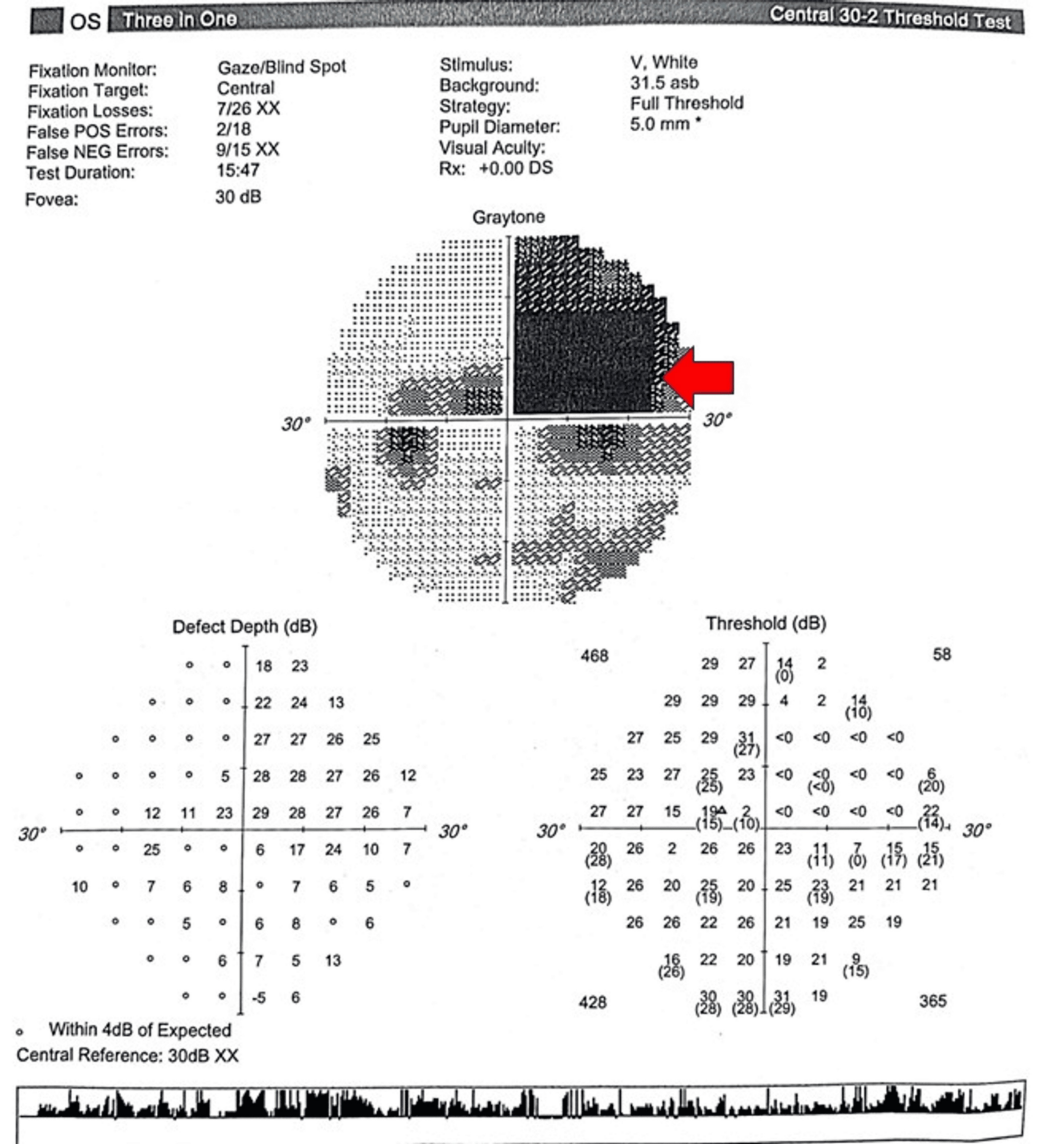
Humphrey Visual Field test showed depression in sensitivity in left eye. POS: false positive error; NEG: false negative error

**Figure 7:**
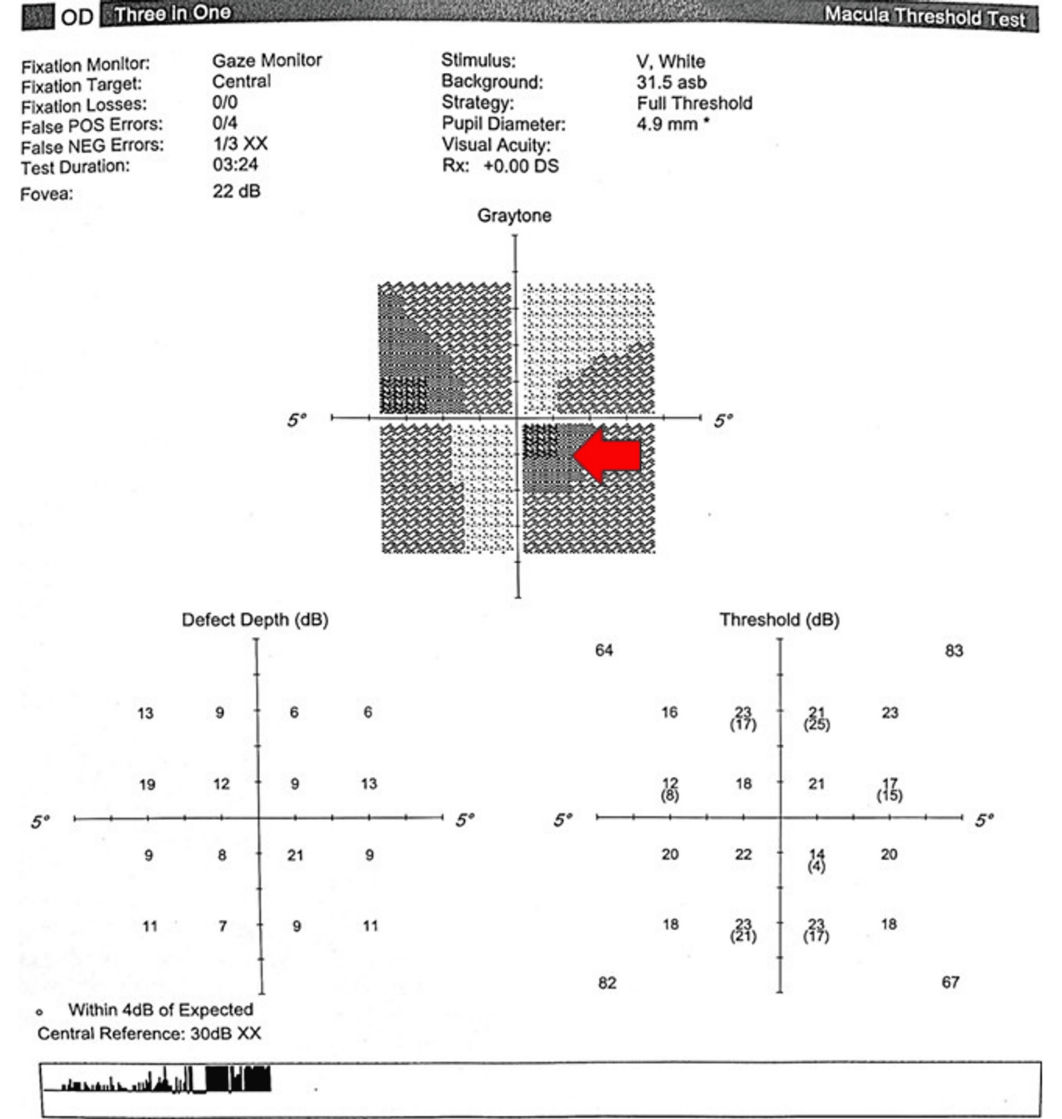
Macular threshold test shows focal depression in right eye. POS: false positive error; NEG: false negative error

**Figure 8:**
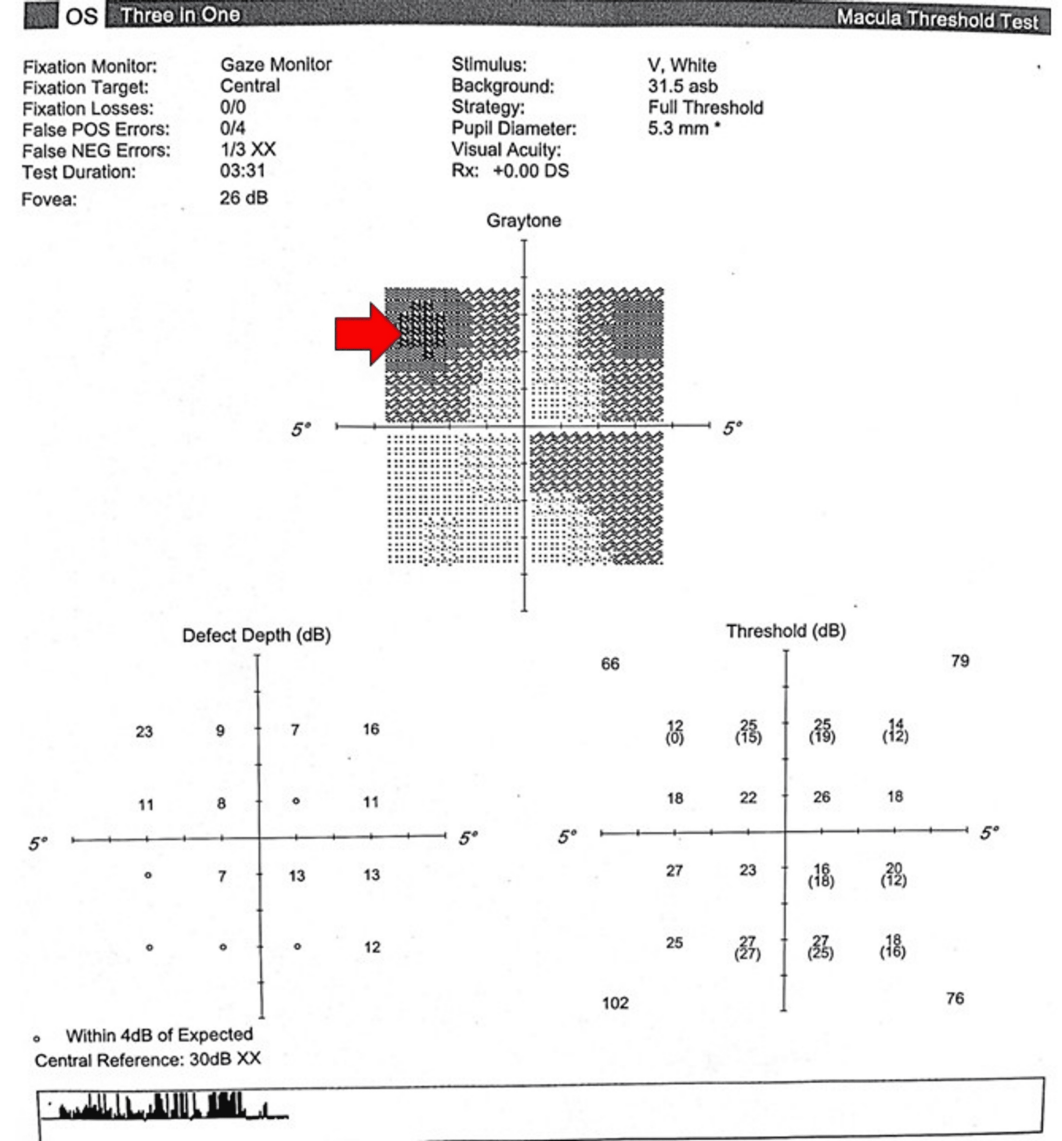
Macular threshold test shows focal depression in left eye. POS: false positive error; NEG: false negative error

Dry retinoscopy values were -1.00 DS / -1.00 DC × 180 in the right eye (OD) and -1.50 DS / -0.50 DC × 20 in the left eye (OS). Cyclo refraction values are OD -0.50 DS / -1.00 DC × 180 and OS -1.00 DS / -0.50 DC × 20. Based on subjective acceptance, the following spectacle prescription was prescribed: OD: -1.00 DS / -0.75 DC × 180 and OS: -1.25 DS / -0.50 DC × 20, which resulted in an improvement in best-corrected visual acuity to OU: 0.86 logMAR. Near visual acuity remained the same as the initial measurement. During the color vision assessment using the Ishihara color vision chart, the patient was able to read only the demonstration plate with both eyes (OU:1/17). No difference was observed using the red filter. The cornea and crystalline lens were clear with no opacities. Fundus examination showed bilateral symmetric atrophic areas in the macula with loss of retinal pigment epithelium (RPE), scattered flecks, RPE alterations, and atrophy, indicating unclassified macular dystrophy in both eyes. Fundus autofluorescence showed hyperautofluorescence at the macula, along with diffuse loss of RPE (Figure 1), and color fundus photography shows bilateral symmetric atrophic areas in the macula (Figure 2). Optical coherence tomography (OCT) revealed thinning of the fovea (OD: 111 μm and OS: 91 μm) with disorganization of the retinal pigment epithelium (Figures 3, 4). Perimetry (central 30-2) (Figures 5, 6) using the Humphrey Visual Field test showed depression in sensitivity at the macula in both eyes, which was identified with more reliability as a focal depression on the macular threshold test (Figures 7, 8). Based on these findings, a diagnosis of unclassified macular dystrophy (atypical) was made, and visual rehabilitation was planned.

**Figure 9:**
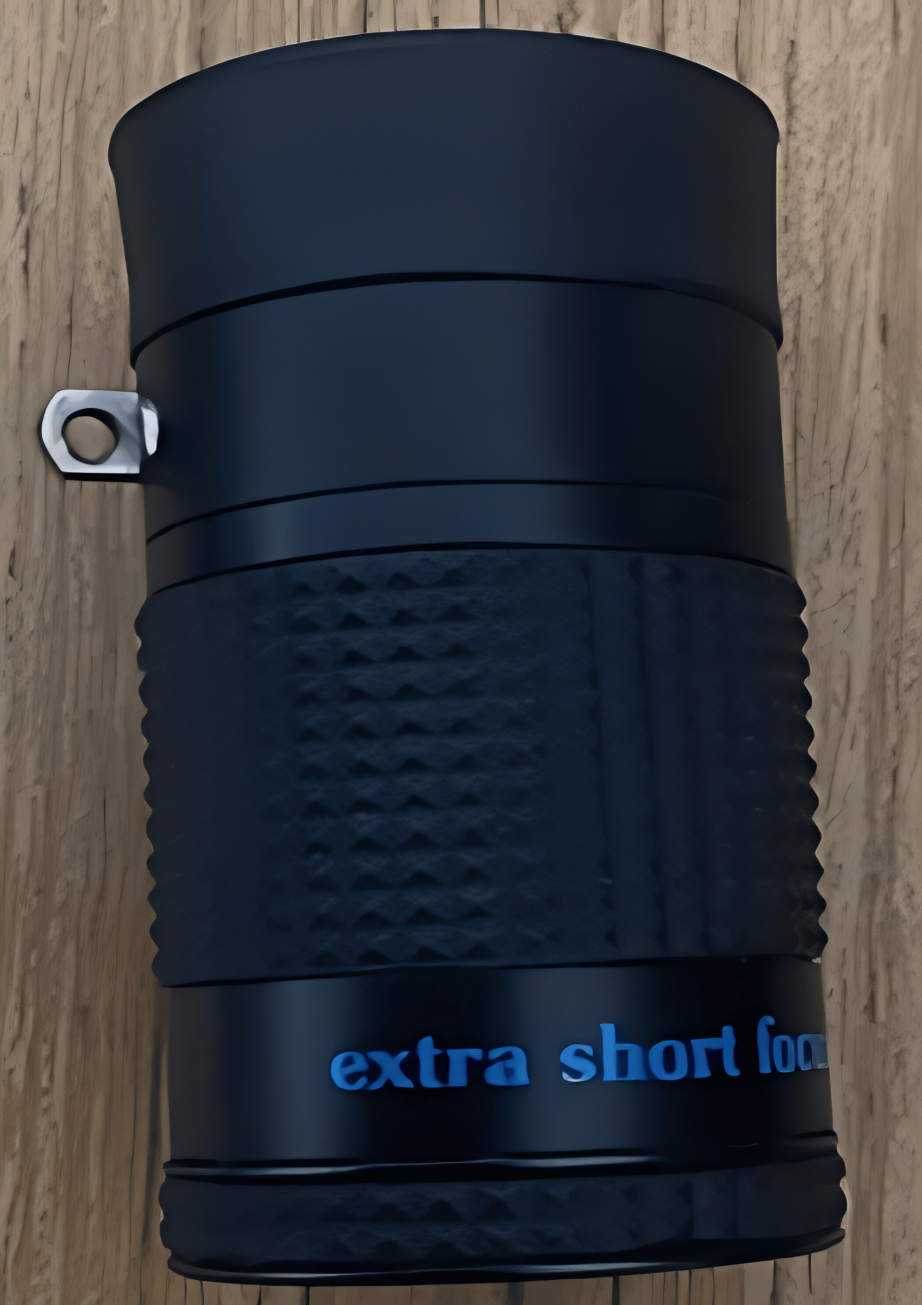
Monocular telescope (4×).

For distance, considering the patient’s foveal threshold and functional visual requirements, a 4× monocular telescope was prescribed for the left eye, resulting in an improvement in visual acuity to 0.5 logMAR (Figure 9). Additionally, eccentric training was provided to help the patient utilize the preferred retinal locus (PRL).

Eccentric viewing training was provided using conventional low-vision rehabilitation techniques and not microperimetry. Eccentric viewing training was provided as part of low-vision rehabilitation to help the patient use a preferred retinal locus (PRL). The patient was trained to shift fixation away from the central scotoma using high-contrast near targets, gradually progressing from single letters to continuous reading. Once a stable PRL was identified, fixation stability and reading efficiency were reinforced. Distance tasks were also included, with emphasis on contrast and optimal illumination. Home practice was advised to support carryover.

**Figure 10:**
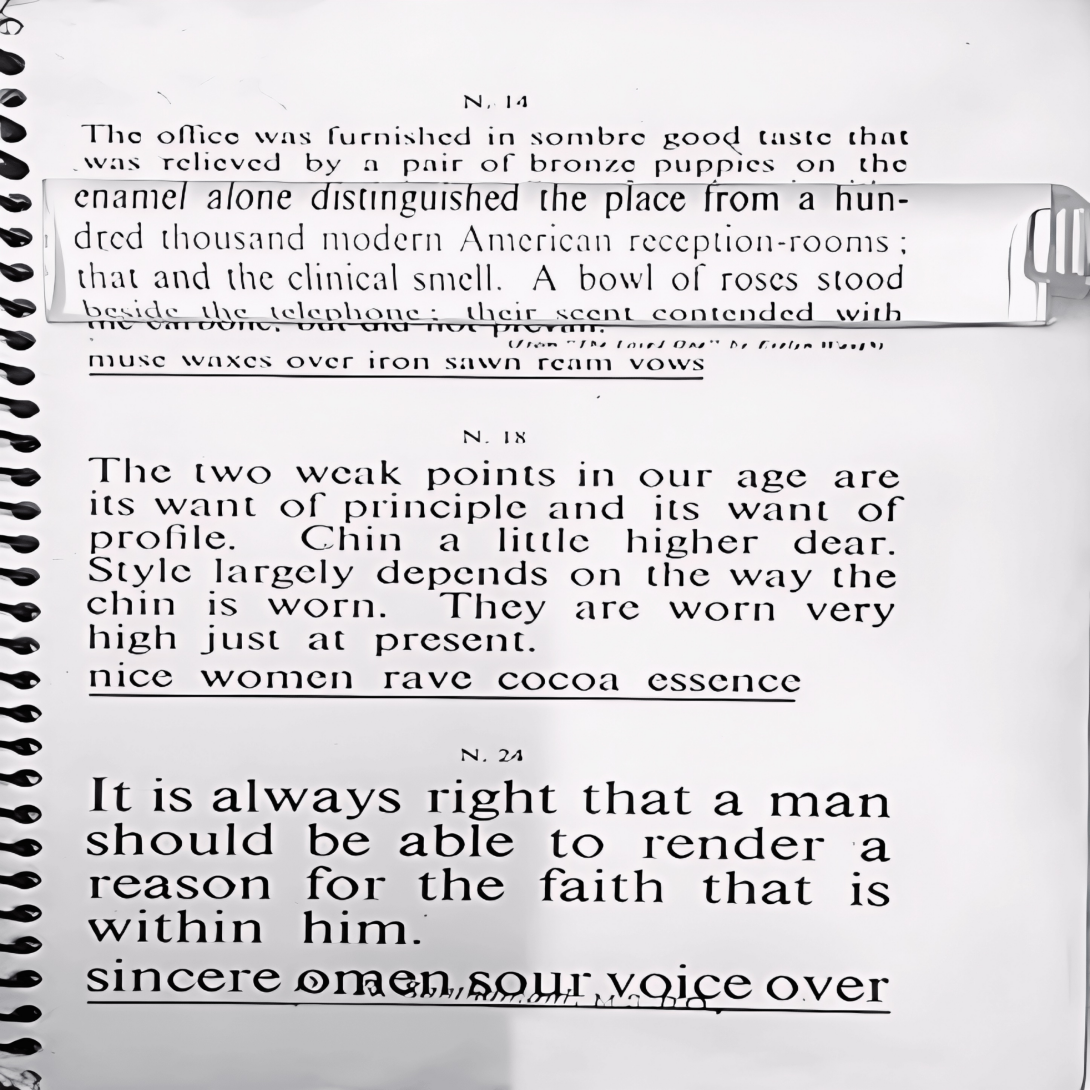
Directory reader (2×) placed on continuous text card.

The following low-vision aids were prescribed immediately following the trial: a directory reader (2×) to be used with adequate direct illumination (1000 lux), high-add lenses (+6D) for near tasks, and a monocular telescope (4×) for distance tasks (Figure 10). Psychological counseling was further advised to support her emotional well-being. 

## Discussion

Macular dystrophy in children presents distinct challenges due to its severe and often irreversible impact on vision, affecting both development and education. Following confirmation of diagnosis of macular dystrophy, it must be further followed up for classification as typical or atypical [10]. Unlike adults, children depend heavily on visual input for learning and social engagement, making early and effective management essential [11]. This study shows the path in addressing low vision in a young girl with partial vision loss and atypical fundus features, thus highlighting the need for a multidisciplinary approach.

Our patient presented with visual impairment characterized by difficulty in reading, writing, and face recognition, which are typical of macular dysfunction. Clinical findings, including absence of the foveal reflex, macular atrophy, and retinal pigment epithelium (RPE) changes, suggested macular dystrophy [1].

A differential diagnosis of Stargardt disease, Best vitelliform dystrophy, and cone dystrophy was considered. Although Stargardt disease commonly presents in childhood with central vision loss, the absence of characteristic pisciform flecks and beaten-bronze appearance made it less likely [1,10]. Best vitelliform dystrophy was excluded due to the absence of vitelliform/vitelliruptive lesions, fibrosis, or the typical “egg-yolk” appearance on fundus examination and OCT [1,4]. Cone dystrophy was also considered because of reduced visual acuity and color vision impairment; however, hallmark symptoms such as marked photophobia and generalized cone dysfunction were not evident. ERG, which could have aided further differentiation, was advised but not completed. Therefore, the atypical clinical and imaging findings supported a diagnosis of unclassified/atypical macular dystrophy [1,12].

At 14 years of age, the patient is within the active progression phase, and the current findings likely represent an evolving phenotype. Puberty-related changes may further influence disease expression; hence, the presentation should be considered part of ongoing progression, warranting longitudinal follow-up. These findings supported a diagnosis of atypical macular dystrophy.

Fundus autofluorescence showed areas of hypoautofluorescence surrounded by hyperautofluorescence, indicating RPE loss with surrounding dysfunction and altered lipofuscin distribution [13]. Macular perimetry revealed reduced sensitivity, evident as focal depression in the macular threshold test [14].

Reduced near vision significantly affected daily and academic activities. While spectacle correction offered minimal benefit, low-vision aids such as a dome magnifier (directory reader) and high-add lenses improved near performance. For distance, based on the foveal threshold and functional needs, a monocular telescope improved visual acuity and enabled better face recognition. Eccentric viewing training further enhanced functional vision by facilitating effective use of a preferred retinal locus [15]. The patient required an adaptation period to become proficient with the devices, including working distance and handling.

This case has certain limitations. The Farnsworth D-15 test was not performed to classify the type of color vision deficiency, and electroretinography (ERG), although advised, was not completed.

## Conclusions

Macular dystrophy in children significantly impacts vision, education, and daily living activities, requiring a comprehensive approach. Early diagnosis and tailored low-vision rehabilitation, including optical aids and eccentric viewing training, can enhance functional vision and academic capabilities. This case underscores the necessity of individualized rehabilitation strategies to optimize residual vision, enhance independence, and improve overall well-being in children with macular dystrophy.
